# The inflammatory response induced by *Pseudomonas aeruginosa* in macrophages enhances apoptotic cell removal

**DOI:** 10.1038/s41598-021-81557-1

**Published:** 2021-01-27

**Authors:** Adriana Valeria Jäger, Paula Arias, Maria Virginia Tribulatti, Marcela Adriana Brocco, Maria Victoria Pepe, Arlinet Kierbel

**Affiliations:** Instituto de Investigaciones Biotecnológicas “Dr. R. Ugalde”, IIBIO, Universidad Nacional de San Martín (UNSAM), CONICET, B1650HMP Buenos Aires, Argentina

**Keywords:** Cell biology, Microbiology, Pathogenesis

## Abstract

Pathogens phagocytosis and the uptake of apoptotic cells (efferocytosis) are essential macrophages tasks, classically considered as mutually exclusive. Macrophages have been observed to polarize into either pro-inflammatory/microbicidal or anti-inflammatory/efferocytic phenotypes. However, macrophage functions have shown to be more complex. Furthermore, little is known about the regulation of efferocytosis under inflammatory conditions. In this study, we elucidate the modulation of the macrophage efferocytic function during an inflammatory stimulus. We find that bone marrow-derived macrophages (BMDM) are very efficient in engulfing both the bacterial pathogen *Pseudomonas aeruginosa* and apoptotic cells. BMDM showed a high bactericidal capacity unaffected by the concomitant presence of apoptotic material. Plasticity in macrophage programming, in response to changing environmental cues, may modulate efferocytic capability. In this work, we further show that, after phagocyting and processing *Pseudomonas aeruginosa,* macrophages highly increase their efferocytic capacity without affecting their phagocytic function. Moreover, we demonstrate that *Pseudomonas aeruginosa* enhances efferocytosis of these phagocytes through the IL-6 signaling pathway. Our results show that the inflammatory response generated by the bacterial processing enhances these macrophages’ capacity to control inflammation through an increased efferocytosis.

## Introduction

Macrophages are key players in both innate and adaptive immunity. They are the first line of defense against pathogens (phagocytosis) and they respond efficiently to tissue injury by removing dead cells and cellular debris (efferocytosis). Through their endocytic functions, they participate in the resolution of inflammation, wound healing, tissue homeostasis (after infection or injury), and antigen presentation^1–3^. According to the inducer or stimuli perceived, macrophages are thought to acquire either a microbicidal/pathogen killing phenotype or an efferocytic/healing one^4,5^. However, during infection, immune cells induce inflammation to promote pathogen killing. In this scenario, macrophages reconcile two opposing functions: clearing the infecting pathogen and repairing the damage suffered by the host tissue. Indeed, macrophages can be the initiators of the inflammatory response and participate in its resolution in a second step, through the regulation of their own profile^4,6^.

In the conventional model of inflammation/resolution, it is supposed that inflammation initiates, peaks and then gradually enters a resolution phase^7^. In recent years, an accumulating body of evidence has challenged this linear model of induction and resolution by showing that pro-resolving molecular pathways are triggered as part of the host response during the inflammatory phase; these results suggest a complex balance between pro- and anti-inflammatory events that start in parallel^8,9^. Resolution is now considered to be a component of the acute inflammation program^10^. While it is known that acute inflammation is set off by external invading pathogens or tissue injury, it is not yet clear which external triggers are sensed by macrophages, thus activating pro-inflammatory and pro-resolving molecular pathways as part of the same initial host response.

The nature of the immune response to dying cells is dependent on the signals these cells expressed or released. Cell progression through the different stages of death occurs with the concomitant exposure of different signals that may modulate their clearance and shape the immune response. But, in general terms, it is considered that apoptotic cell uptake triggers anti-inflammatory signaling^11^.

In the context of many infections, there is an inflammatory environment in which bacteria and dead cells coexist as constant stimuli. This is the case of patients with cystic fibrosis, who suffer from chronic lung infections (particularly with *Pseudomonas aeruginosa*) and whose airways harbor an exacerbated number of apoptotic cells^12^. Other contexts are wounds and burns, where *P. aeruginosa* finds dead cells on which to adhere^13^. Furthermore, we have demonstrated that when *P. aeruginosa* infects the epithelial barrier it binds almost exclusively to extruded apoptotic cells. These bacteria-laden apoptotic cells are efferocytosed into epithelial cells where bacteria die^14^. Therefore, these joint stimuli (bacteria and apoptotic cells) confront phagocytes to the concomitant processes of phagocytosis and efferocytosis.

*Pseudomonas aeruginosa* is an opportunistic pathogen that primarily infects immuno-compromised individuals and/or patients with epithelial injury. While the epithelium provides a physical barrier against this gram-negative pathogen, innate immunity and, specifically, phagocytosis by neutrophils and macrophages are key determinants in the ability of the host to control *P. aeruginosa* infection. Thus, the host inflammatory response is intimately connected to the phagocytic clearance of the bacteria.

As signals in the inflammatory milieu may affect the programming of macrophages modulating their endocytic functions, in this research, we further investigate the outcomes for macrophage phagocytic and efferocytic capacities when they (jointly or separated) process apoptotic cells and *Pseudomonas aeruginosa*.

Our results show that, when confronted with this pathogen, macrophages respond through a pro-inflammatory response and microbicidal action to destroy the infectious agent. However, at the same time, the increase of a pro-inflammatory response following the bacterial processing promotes the clearance of apoptotic cells by these macrophages and contributes to the resolution of local inflammation.

## Results

### *Pseudomonas aeruginosa* adheres to apoptotic cells and is internalized by macrophages

The aim of this study was to examine the processing of *P. aeruginosa* strain K (PAK) and apoptotic cells, individually or concurrently, in macrophages and the consequent responses to these different stimuli. For this purpose, primary bone marrow-derived macrophages (BMDM) and J774 macrophage-like cell line (J774A.1), both from BALB/c mice, were used.

We have previously shown that *P. aeruginosa* adheres and forms aggregates onto apoptotic cells spontaneously extruded from monolayers of polarized epithelial cells. We have also shown that apoptotic cells generated by UV irradiation interact with *P. aeruginosa*^14^.

CFSE-permanently-labelled apoptotic J774 cells were used as targets for the BMDM. Our protocol for apoptosis induction resulted in a majority of late apoptotic cells (Supplementary Fig. S1a). The high percentage of cells in this stage is desirable for our studies since, as we previously reported, *P. aeruginosa* preferentially binds to epithelial cells in this apoptotic stage^14^. We verified that this apoptotic population of J774 also adheres bacteria (Supplementary Fig. S1b).

**Figure 1:**
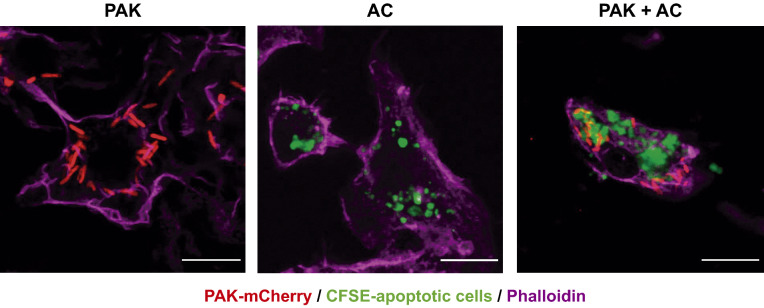
*P. aeruginosa* adheres to J774 apoptotic cells and both are internalized by macrophages. Projected confocal Z-stacks of BMDM exposed for 30 min to bacteria, apoptotic cells, or both. Scale bars 10 µm. Green: CFSE-labeled J774 apoptotic cells. Red: PAK-mCherry. Magenta: phalloidin.

When primary BMDM were exposed to apoptotic cells, bacteria, and bacteria-laden apoptotic cells, they efficiently internalized bacteria (phagocytosis) and apoptotic material (efferocytosis), both independently and together (Fig. 1). In this experimental setup, we obtained cellular populations in which virtually all macrophages phagocyted and/or efferocyted the offered targets. We will refer to the Phagocytic Index as the mean number of bacteria that were ingested per macrophage, and to Efferocytic Index as the average volume of apoptotic corpses that was engulfed per macrophage. Both indexes are expressed as percentages of the corresponding controls (100%). When bacteria-laden apoptotic cells were added as targets, both Phagocytic and Efferocytic Indexes were calculated for the same macrophage population. In terms of absolute numbers, the average phagocytic capacity per BMDM was 5.3 ± 3.9 bacteria; and the efferocytic capacity was 135 ± 120 μm^3^. The volume of internalized material was determined by confocal microscopy and subsequent image analysis which then permitted the calculation of the Phagocytic and Efferocytic Indexes (Methods and Supplementary Fig. S2)^14,15^.

### *Pseudomonas aeruginosa* is rapidly eliminated by macrophages, independent of efferocytosis

There are conflicting results within the literature (see “Discussion”) regarding the survival capacity of *P. aeruginosa* inside macrophage cells. Given this, we evaluated the intracellular survival of *P. aeruginosa* (PAK) in naïve BMDM.

**Figure 2:**
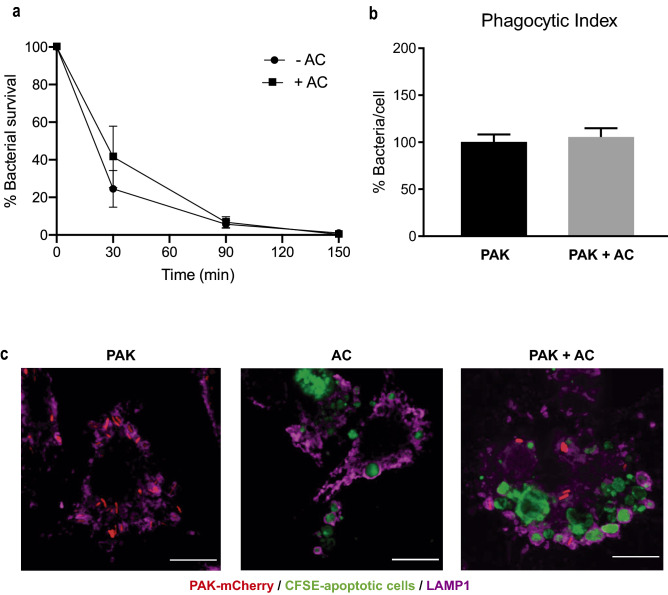
*P. aeruginosa* is eliminated by murine primary BMDM inside LAMP1-positive vesicles. (**a**) Intracellular bacterial survival over time was measured by standard antibiotic protection assays followed by CFU counting. Data presented as mean of CFUs ± SEM were normalized to time 0 (100%). This assay was done in the absence (−AC) or presence of apoptotic cells (+AC). Three independent experiments were performed. No significant differences were found by unpaired *t* test. (**b**) Phagocytic Index. Cells were incubated with the targets for 30 min. The index was calculated as the average number of bacteria internalized per cell in the presence or absence of apoptotic cells. Data were calculated as mean ± SEM and normalized to the condition with only bacteria (100%). *PAK* bacteria alone, *PAK + AC* PAK with apoptotic cells concurrently. Three independent experiments were performed. No significant differences were found by unpaired *t* test. (**c**) *P. aeruginosa* and apoptotic cells localize to LAMP1-positive vesicles. BMDM were fed for 50 min with PAK-mCherry, CFSE-labeled apoptotic cells, or both concurrently. Confocal projected Z-stacks showing LAMP1-positive vesicles containing bacteria and apoptotic material. Scale bars 10 µm. Green: CFSE-labeled apoptotic cells. Red: PAK-mCherry. Magenta: LAMP1.

Intracellular bacterial survival over time was measured by a standard antibiotic protection assay (Fig. 2a). BMDM killed *P. aeruginosa* rapidly: the number of intracellular bacteria decreased to ~ 25% within the first 30 min and to 5% at 90 min. It is worth mentioning that a microscopic examination of the macrophages showed proper morphology and no signs of lysis after bacterial infection. In fact, macrophages were in good morphological and functional condition 18 h after bacterial infection (see next section). A similar intracellular killing pattern was observed for J774 macrophages (Supplementary Fig. S3), supporting the fact that macrophages efficiently kill *P. aeruginosa*. Primary macrophages appeared to be faster in terms of killing.

Apoptotic cell recognition that occurs in parallel with the engagement of PAMP receptors may activate an intricate net of signaling pathways. Since efferocytosis and phagocytosis can occur concomitantly in macrophages when apoptotic cells and *P. aeruginosa* are offered together (Fig. 1), we wondered whether efferocytosis may influence the concurrent bacterial uptake or bactericidal activity shown by these macrophages. For this purpose, we determined the Phagocytic Index with *P. aeruginosa* alone or in concurrence with apoptotic cells. We found that the concomitant presence of apoptotic cells did not change the bacterial uptake ability of these phagocytes (Fig. 2b) (assessed by *t* test). Next, we investigated the bactericidal activity of naïve BMDM challenged with these bacteria-laden apoptotic cells for different periods of time (as described above). Figure 2a shows that the killing rate (% of internalized bacteria killed over time) was similar in the presence or absence of apoptotic cell material (+ AC/− AC), indicating that the concomitant presence of apoptotic cells did not change the bactericidal activity of these phagocytes either (assessed by *t* test).

Moreover, we found that the intracellular localization of bacterial cells and apoptotic debris within macrophages are Lysosomal-associated membrane protein 1 (LAMP1)-positive vesicles (Fig. 2c), suggesting that their destination is the elimination by the lysosomal machinery.

From these results, we conclude that BMDM efficiently uptake and kill *P. aeruginosa,* independent of the conjoint presence of apoptotic cells.

### *Pseudomonas aeruginosa* enhances the efferocytic capacity of BMDM

We reasoned that a pre-incubation of the BMDM with the different stimuli might give these immune cells the required time to activate long term signaling pathways that could translate into changes in their efferocytic and phagocytic capacities. To this end, we pre-incubated the cells for 50 min with different potential inducers: bacteria (PAK), apoptotic cells (AC), PAK-laden AC (PAK + AC), and the Control (no inducer) (see Methods). The corresponding targets were offered after 18 h. These four differently-induced macrophage populations were challenged with the two targets of interest, AC and PAK. The Efferocytic and Phagocytic Indexes were then calculated.

**Figure 3:**
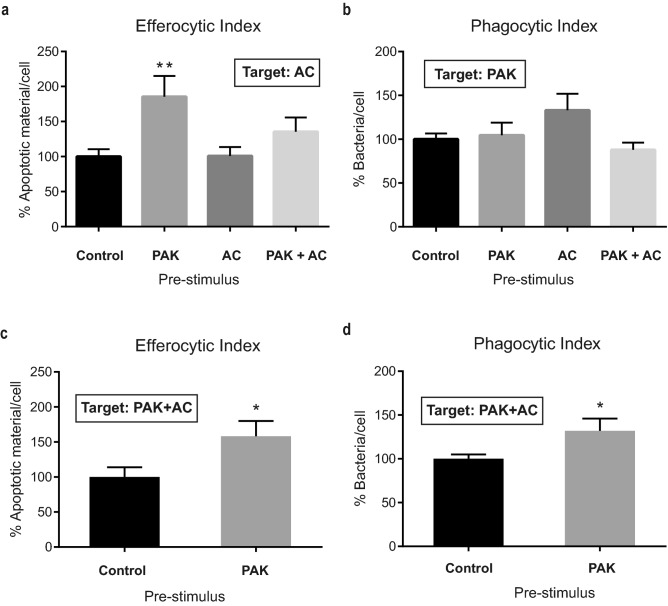
BMDM Efferocytic Index is increased by *P. aeruginosa* pre-stimulation*.* (**a**) BMDM Efferocytic Indexes after different stimuli are shown. BMDM were incubated for 50 min with different pre-stimuli: PAK (bacteria), AC (apoptotic cells), PAK + AC (PAK with AC concurrently) and Control (non-stimulus). The efferocytic capacities using apoptotic cells as targets were checked 18 h after pre-stimuli. The Phagocytic Indexes for these same macrophage populations were calculated and are shown in panel (**b**). Analysis of BMDM efferocytic (**c**) and phagocytic (**d**) Indexes after pre-stimulation with bacteria (or non-stimulated as Control) and confronted with bacteria-laden apoptotic cells as targets. Same experimental conditions as in (**a**,**b**) but using PAK and AC concurrently as targets for the assays. Data were normalized to the Control (non-stimulus: 100%) and expressed as mean of apoptotic material/cell or bacteria /cell ± SEM of three independent experiments. In (**a**) **p = 0.0098 *vs.* Control by one-way ANOVA, followed by Dunnett´s multiple comparison test. In (**c**,**d**) * p < 0.05 *vs.* Control by unpaired *t* test.

When analyzing the efferocytic ability of pre-stimulated macrophages a remarkable finding emerged. Macrophages that had been previously exposed to PAK as a pre-stimulus showed a significant rise in their efferocytic function (Fig. 3a). Notably, this effect was seen with PAK as unique pre-stimulus, and not in combination with apoptotic cells, suggesting a possible antagonistic effect of these cells.

Regarding the phagocytic process, no differences were found among the different pre-stimuli (Fig. 3b) (with these experimental settings LPS causes an 80% rise in phagocytosis, as shown in Supplementary Fig. S4b).

As mentioned, there are several pathological circumstances in which a persistent bacterial infection simultaneously occurs with an exacerbation of the presence of dead cells. Within these contexts, immune cells are exposed to both stimuli: bacteria and apoptotic cells. In this study, we have shown that PAK and AC can bind together (Supplementary Fig. S1b). This fact, together with the finding that bacteria enhance efferocytosis (Fig. 3a) prompted us to investigate the outcome regarding the clearance capacity of PAK-pre-stimulated macrophages when the targets offered were bacteria-laden apoptotic cells instead. In this context, macrophages also showed an increased Efferocytic Index when pre-stimulated with PAK: a 58% rise with respect to unstimulated control (p < 0.05) (Fig. 3c). This result is in line with our previous findings: in both assays, efferocytosis augmented after PAK pre-stimulus whether targets were AC alone or bacteria-laden AC. These results clearly show that the prior stimulus with bacteria enhances the efferocytic capacities of BMDM. Furthermore, we have also analyzed the Phagocytic Index to elucidate if the improved engulfment of apoptotic cells results in a concomitant uptake of the associated bacteria. As shown in Fig. 3d there was an increase of 32% in the Phagocytic Index after bacterial challenging of the BMDM (p < 0.05) when bacteria-laden apoptotic cells were offered as targets.

### *Pseudomonas aeruginosa* alone or in conjunction with apoptotic cells elicits a strong cytokine response in BMDM

**Figure 4:**
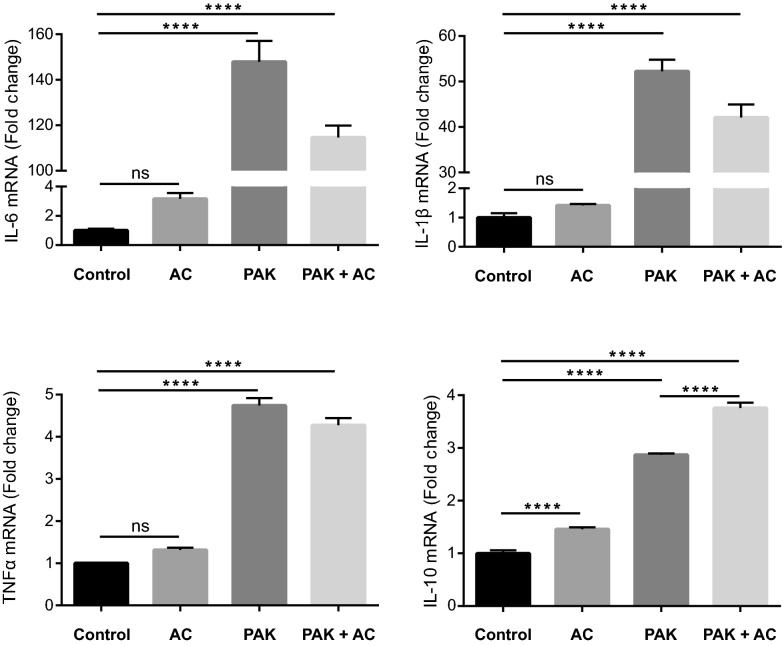
BMDM cytokine gene expression profile elicited by the different stimuli. Cytokine gene expression was determined in BMDM after different pre-stimuli treatment and expressed as a fold change with respect to Control. BMDM were incubated for 50 min with different pre-stimuli: AC (apoptotic cells), PAK (bacteria), PAK + AC (PAK with AC concurrently) and Control (non-stimulus). Six hours after the initiation of the experiment, RNA was extracted. Four mRNA cytokines were tested by RT-qPCR: *Il-6*, *Il-1β*, *Tnfα* and *Il-10*. Data is presented as mean Fold Change ± SD. One of three independent experiments normalized to *Ywhaz* mRNA levels is shown. ****p < 0.0001 *vs.* Control by one-way ANOVA, followed by Tukey’s multiple comparison test.

Macrophages are cells with high plasticity that respond to different environmental stimuli with the release of cytokines. Bacteria, apoptotic cells, or the combination of both may affect the cytokine secretion profile of the engulfing macrophages differently, modulating their functional phenotype. We measured changes in cytokine expression levels in BMDM by real-time RT-PCR. BMDM were exposed to the different stimuli (or non-stimulus, as Control) for 50 min: *P. aeruginosa* (PAK), apoptotic cells (AC), or bacteria-laden apoptotic cells (PAK + AC) (see Methods). Six hours after the experiment’s initiation, the total RNA was extracted, and poly(A) RNA was reverse transcribed for subsequent SYBR Green qPCR. The selected cytokines were: IL-6, IL-1β, and TNFα as classically described pro-inflammatory and IL-10 as a typical anti-inflammatory. In general, we observed that *P. aeruginosa* and *P. aeruginosa*-laden apoptotic cells generated the strongest differences in cytokine expression compared to the untreated cells (Control) (Fig. 4). *IL-6* and *IL-1β* mRNAs showed the highest levels of expression and the strongest increases due to bacteria stimulation, regardless of the presence of apoptotic cells. For TNFα and IL-10 there was also an increase in their gene expression with PAK stimulus and with PAK + AC.

Bacteria generated a very strong response in BMDM. After pathogen recognition, high levels of pro-inflammatory cytokine (IL-6, IL-1β, and TNFα) mRNA were produced. An increase in the gene expression of the anti-inflammatory cytokine IL-10 can also be observed; other studies have reported a similar rise after the increase of the inflammatory cytokine IL-6. IL-6 is described as a pro-inflammatory mediator and an anti-inflammation regulator stimulating anti-inflammatory cytokines^16^. Subsequently, IL-10 is expected to limit the production of pro-inflammatory cytokines (including IL-6, IL-1β, and TNFα) (reviewed in^17^).

As for apoptotic cells alone as stimulus, there was a significant increase in IL-10 cytokine gene expression (Fig. 4) as previously shown in the macrophage-like cell line RAW^18^. Remarkably, PAK-laden apoptotic cells induced a stronger and statistically significant increase in *Il-10* mRNA levels with respect to macrophages treated with PAK alone. This was not the case for the other three tested cytokines, where no significant differences between the stimuli (PAK and PAK + AC) were consistently detected. Recent studies have suggested that apoptotic cells inhibit macrophage responses as an anti-inflammatory signal^19^. Authors have shown that IL-6 and TNFα secretion by LPS-activated macrophages is abrogated after efferocytosis of apoptotic targets, indicating that apoptotic cells actively antagonize pro-inflammatory signals. As mentioned, when we stimulated the macrophages with PAK + AC, the pro-inflammatory cytokine expression still increased. However, the rise in the anti-inflammatory cytokine IL-10 was significantly higher (with respect to PAK alone as pre-stimulus). On the other hand, it is worth mentioning that, in our experimental settings, PAK as a pro-inflammatory stimulus generated stronger cytokine increases than LPS (Supplementary Fig. S4c).

### IL-6 mediates the increased efferocytic capacity of bacterial-stimulated BMDM

It has been recently reported that activation of the STAT3-IL-10-IL-6 pathway in BMDM leads to the positive regulation of macrophage efferocytosis^20^. In this study, the authors showed that IL-6 production is important for efferocytosis and for the phenotypic switch of the macrophages from inflammatory to restorative. Furthermore, IL-6 has been shown to enhance the efferocytic capacity of human macrophages^21^.

**Figure 5:**
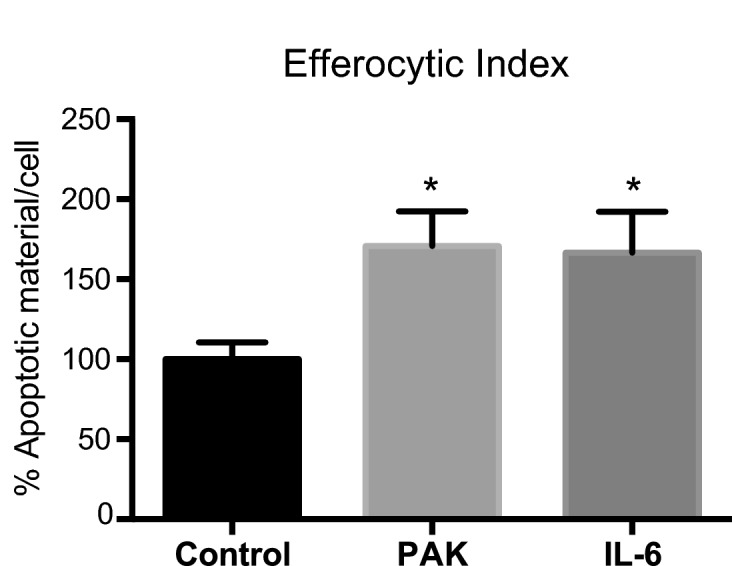
BMDM Efferocytic Index is increased by IL-6 pre-stimulation. BMDM Efferocytic Index after pre-stimulation with PAK or IL-6 (10 ng/ml). BMDM (from C57BL/6J mice) were incubated for 50 min with PAK or IL-6, and non-stimulus as Control. The efferocytic capacities using apoptotic cells as targets were checked 18 h after pre-stimuli. Data were normalized to the Control (non-stimulus: 100%) and expressed as mean of apoptotic material/cell ± SEM of two independent experiments. *p < 0.03 *vs.* Control by unpaired *t* test.

From our results, we know that PAK enhances the efferocytic capacity of BMDM (Fig. 3a,c). Also, these bacteria cause a strong response of IL-6 expression (Fig. 4). Despite being commonly associated with pro-inflammatory functions, IL-6 can enhance the polarization of anti-inflammatory or alternatively activated macrophages^22^. Therefore, we next wondered whether the IL-6 upregulation was linked to the observed increase in the efferocytic capabilities of these cells. For this purpose, we directly assessed the effect of this cytokine on macrophages’ efferocytic capacity by pre-stimulating them with recombinant IL-6. As shown in Fig. 5, macrophages that have been previously exposed to IL-6 as a pre-stimulus showed a significant increase in their efferocytic function when confronted with apoptotic cells as targets. Moreover, they increased efferocytosis to the same levels as macrophages exposed to PAK (this experiment was performed in BMDM differentiated from C57BL/6J mice, also corroborating the observed increase in efferocytosis in another mouse strain).

**Figure 6:**
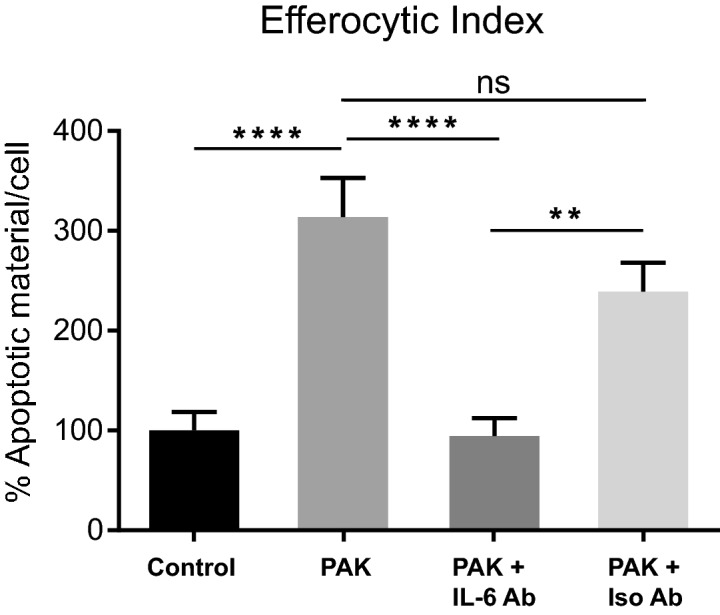
IL-6 mediates the increase of the Efferocytic Index in bacterial pre-stimulated BMDM. BMDM were incubated for 50 min with PAK (bacteria) as pre-stimulus or left unstimulated (Control). The efferocytic capacities using apoptotic cells as targets were checked 18 h after pre-stimuli. Additionally, PAK pre-treatment was evaluated in the constant presence of anti-IL-6-neutralizing monoclonal antibody (IL-6 Ab) or its matching isotype Control (Iso Ab). Efferocytic capacity was determined by confocal microscopy as detailed in Methods. Data were normalized to the Control (non-stimulus: 100%) and expressed as mean ± SEM of three independent experiments, **p = 0.0019 and ****p < 0.0001 *vs.* Control by one-way ANOVA, followed by Tukey’s multiple comparison test.

Next, we specifically analyzed the relative contribution of IL-6 to the enhanced efferocytic capabilities of these macrophages. To this, we used a specific antibody to neutralize the potential IL-6 effect on the augmentation of efferocytosis after bacterial stimuli of the macrophages. As seen in Fig. 3a, cells were pre-stimulated or not (Control) with bacteria (PAK) for 50 min and incubated for 18 h before proceeding with the efferocytosis assay (using apoptotic cells as targets). In this experiment, we evaluated the PAK pre-treatment condition in the constant presence of anti-IL-6-neutralizing monoclonal (IL-6 Ab) or its isotype control (Iso Ab) antibodies. We observed that the Efferocytic Index of PAK pre-stimulated macrophages treated with anti-IL-6 decreased to control cell level (Fig. 6, p < 0.0001). On the other hand, the efferocytic capacity of bacterial-stimulated cells was not affected in the presence of the corresponding isotype control, thus confirming the specificity of this effect. These results indicate that IL-6 mediates the PAK-induced efferocytosis observed in BMDM.

## Discussion

Bacterial killing is complex and depends on the bacterial strain, environmental conditions, and cell type/tissue^23^. The literature on this subject offers contradictory evidence regarding the survival capacity of different strains of *P. aeruginosa* inside different types of macrophages^23–26^. Our results show that BMDM and J774 macrophages can kill *P. aeruginosa* very efficiently. Also, bacteria were found inside LAMP1-positive vesicles, suggesting their final elimination inside lysosomes. Notably, the processing of bacteria seems to transform macrophages into a pro-efferocytic phenotype, while retaining (Fig. 3b) or even enhancing (Fig. 3d) their phagocytic capacity.

To further investigate the functional changes that *P. aeruginosa* induces in these macrophage cells, we have analyzed cytokine mRNA expression changes in BMDM upon interaction with different stimuli. Bacteria elicited a strong pro-inflammatory profile, where IL-6 was the most upregulated cytokine. Notably, IL-6 resulted in a key mediator for the augmented efferocytic capacity observed in bacteria pre-stimulated macrophages.

An established concept is that macrophage recognition of apoptotic cells can shift their programming toward a pro-resolving phenotype. This involves the production of several modulators (TGFβ and IL-10), which in turn enhance the resolution of inflammation and block the release of pro-inflammatory mediators^27–30^. In our experimental model which examines the effect of late apoptotic cells as targets (previous studies mostly used early apoptotic cells as targets for the efferocytic assays), we have also observed an increase in IL-10 expression when apoptotic cells were given as a stimulus alone or in concurrence with bacteria. But we did not see other pro-resolving outcomes: when apoptotic cells alone were used as pre-stimulus, we did not observe a change in the efferocytic capacity (Fig. 3a). It has been shown that phosphatidylserine (which is externalized to the outer membrane leaflet of apoptotic cells) reversed impaired macrophage efferocytosis in an in vivo model of chronic granulomatous disease, but in that case, *wild-type* macrophages were unaffected^31^. Specifically, for the process of efferocytosis, the literature has yet to indicate whether the uptake of a dying cell affects the ability to uptake other dead cells^30^.

When further analyzing the efferocytic capacity of macrophages under concomitant pre-stimulation with bacteria and apoptotic cells (Fig. 3a), we found that apoptotic cells prevented the increase in the Efferocytic Index that occurs when bacteria are present as a unique pre-stimulus. This discrepancy in the resulting Efferocytic Indexes occurs even though both pre-stimulation conditions (bacteria alone and bacteria plus apoptotic cells) have similar tested cytokine expression profiles. In particular, they both revealed a strong increase in IL-6. This may indicate that under the conjoint stimuli, the high IL-6 expression may not be enough to sustain an increased efferocytic capacity in macrophages. This suggests that the signaling elicited by the apoptotic cells may generate an antagonistic effect. This result requires further investigation and highlights the complexity of efferocytosis regulation when multiple stimuli are in play.

There is abundant evidence that the failure to clear apoptotic cells exacerbates inflammation in vivo, indicating efferocytosis plays a crucial role in promoting resolution^32–35^. In this work, we further show that the proinflammatory response generated by macrophages after bacterial stimulus induces an upregulation of the clearance of apoptotic cells. Therefore, this mechanism might be very relevant in the context of infection/inflammation of tissues, where efferocytosis is needed for the ultimate resolution of local inflammation.

We propose that IL-6 acts as a principal mediator in the mechanism that links the inflammatory response against a pathogen, to the resolution of inflammation through efferocytosis. Moreover, since *P. aeruginosa* adheres to apoptotic cells, the increased efferocytosis could further help to resolve the infection.

Although IL-6 is primarily considered a major pro-inflammatory cytokine, it may also act as a key anti-inflammatory mediator in the control of inflammatory responses^36^. Data supporting this concept is continuously emerging and our work makes a relevant contribution by proposing that IL-6 acts as an initiator of resolution, triggered during the inflammatory phase of infection.

Previous studies have reported the induction of efferocytosis by IL-6 in human macrophages^21^. In this work, Frisdal et al*.* illustrated that the addition of recombinant IL-6 generates an increase in the Efferocytic Index and an increase in the gene expression of the efferocytic receptor MERTK. They also observed that this cytokine attenuates the pro-inflammatory human macrophage response as sustained stimulation with IL-6 led, for example, to an elevation of IL-10. In our experimental settings, we have also seen an increase in IL-10 gene expression because of bacterial stimulation, which was even stronger when bacteria and apoptotic cells were tested as joint stimuli. In addition, research from Campana et al*.* suggests that IL-6 is sufficient to increase efferocytosis in vivo and to promote macrophages to switch from an inflammatory phenotype to a restorative one^20^.

As described, IL-6 undergoes an autocrine action that promotes the clearance of apoptotic cells. Our research provides evidence of this, but also goes a step further by describing that a pathogen can be the trigger for the IL-6 mediated upregulation of efferocytosis. In a bacterial infectious context, *P. aeruginosa* might act as a mediator for the macrophage phenotype switch towards a pro-restorative phenotype, required for inflammation resolution. This is particularly interesting given that bacterial infections are mainly described as triggers for a canonical type 1 response. Along these lines, Mukherjee et al*.* describe how the Bacillus Calmette-Guerin (BCG) enhances efferocytosis by alveolar phagocytes *in vivo*^37^. In the context of *Listeria monocytogenes* liver infection and by way of a different mechanism, Bleriot et al*.* describe that M1 and M2 phenotypes (classically considered to be mutually exclusive) are triggered by the pathogen in a consecutive and complementary manner, rather than by a phenotype switch^38^. These studies highlight the role that the microenvironment plays in determining macrophage fate, to efficiently limit tissue responses to infection, and proceed toward tissue repair.

Studies have illustrated that inflammatory stimuli cause the upregulation of Axl (a TAM receptor), which is involved in efferocytosis on murine and human macrophages^39–41^. Specifically, the induction of Axl by inflammatory stimuli associated with viral infections is involved in maintaining the human macrophage efferocytic capacity, which may have important consequences for the resolution of anti-viral immune responses^40^. It would be interesting to test for possible regulation of efferocytic receptors by *P. aeruginosa* in our experimental system.

Macrophages interpret signals from the environment and coordinate an early pro-inflammatory response to pathogens and tissue injury; they ultimately produce anti-inflammatory intermediaries for the active suppression of inflammation and restoration of tissue homeostasis. Removal of apoptotic cells is crucial for these latter processes. Besides being a disposal mechanism that gets rid of dying cells and limits the release of intracellular damage-associated molecular patterns, efferocytosis has been shown to be capable of actively shaping immune responses and influencing tissue restorative programs. Emerging studies describe efferocytosis as a common target for pathogens toward the resolution of infection. Now, the challenge is to begin to understand how the spatiotemporal dynamics and potential relationships among the various signaling pathways that are activated in the context of infection affect cell clearance and immunity. Given this, our results provide evidence for a relationship between *P. aeruginosa* infection of macrophages and their ability to remove dying cells.

## Methods

### Reagents

Chemical and protein reagents used (and vendors) were as follows: Anti-LAMP1 antibody 1D4B: DSHB (Iowa City, IA, USA). Alexa-fluor 647 goat-αRat IgG and CFSE (Carboxyfluorescein succinimydil ester): Invitrogen (Waltham, MA, USA). Alexa-fluor 647 conjugated phalloidin and Sytox Green nucleic acid stain: Life Technologies (Carlsbad, CA, USA). Cell culture medium RPMI 1640: Gibco. Recombinant murine M-CSF: PeproTech (NJ, USA). Recombinant mouse IL-6, PE anti-mouse CD11b, Ultra-LEAF purified anti-mouse IL-6 (clone MP5-20F3) and Ultra-LEAF purified rat IgG1, κ Isotype Control antibodies: Biolegend (San Diego, CA, USA).

### Cell culture

Murine macrophage (J774A.1) and bone marrow-derived macrophages (BMDM) from BALB/c mice were routinely cultured at 37 °C with 5% CO_2_ in RPMI Complete Medium: RPMI 1640 supplemented with 10% FBS, 1% antibiotic–antimycotic, 2 mM sodium pyruvate and 2 mM l-glutamine. Where indicated, BMDM from C57BL/6J mice were used instead. All procedures requiring animals were performed in agreement with institutional guidelines and approved by the Ethics Committee for the Care and Use of Experimental Animals of the Universidad Nacional de San Martín (CICUAE-UNSAM).

### BMDM preparation

Fresh bone marrow cells were prepared by standard techniques^42^. Cells were resuspended in 10 ml of RPMI Complete Medium supplemented with 20 ng/ml of M-CSF and seeded in 100-mm culture dishes. At day 3 and 6, half of the medium was changed with fresh supplemented RPMI. At day 8 macrophages were detached by gently scrapping with ice-cold PBS. Flow cytometry evaluation showed that more than 98% of cells were CD11b-positive. Expression of CD86 and MHC-II on naïve or LPS-stimulated CD11b-positive BMDM was analyzed by flow cytometry (Supplementary Fig. S4a). As expected, LPS stimulation induced the up-regulation of both surface molecules.

### Generation of apoptotic cells (AC)

Confluent J774 cells were resuspended in serum-free RPMI. At this point, cells were labelled (if needed) with 2.5 µM of the fluorescent molecule CFSE. They were seeded on 100-mm culture plates and treated with short-wave UV (50 mJ/cm^2^) in a UVP CL-100 Ultraviolet Crosslinker, for 1 min. Afterwards, cells were incubated for 16 h in serum-free media. Non-adherent AC were harvested from the supernatants by centrifugation (300*g* for 5 min) and apoptosis was assessed by flow cytometry. For this purpose, cells were resuspended with 100 nM of Sytox Green for nucleic acid staining, and Alexa 647 conjugated-Annexin V (1/20) for phosphatidylserine externalization detection. A negative control was performed using live cells that were trypsinized and stained with both reagents. Analysis was performed in a FlowMax cytometer PASIII (Partec, Münster, Germany). The percentages of differently labeled cells were calculated with FlowJo Software (Tree Star Inc., OR, USA). This protocol resulted in 22% of early apoptotic cells (AnnexinV-Alexa-647 single-labeled cells), and 62% of late apoptotic cells (AnnexinV-Alexa647 / SytoxGreen double-labeled cells) (Supplementary Fig. S1a). Bacteria adherence to this apoptotic population of J774 was corroborated by incubation with *P. aeruginosa-*mCherry for 1 h (Supplementary Fig. S1b). In this study, we used a ratio 3:1 of AC:viable macrophages.

### Bacterial culture

*Pseudomonas aeruginosa* strain K (PAK) was routinely grown shaking overnight in LB at 37 °C. Stationary-phase bacteria were used for co-incubation with macrophages. For fluorescence microscopy studies, PAK carrying a plasmid containing the mCherry gene was used (pMP7605 plasmid was generously gifted by Dr. Lagendijk^43^). In this study, we used a MOI of 40 in all the experiments performed with BMDM. When bacteria and apoptotic cells were offered conjointly to macrophages, we included a pre-incubation step for these two targets (co-incubation) for 50 min at 37 °C in RPMI medium before adding them to the phagocytes.

### Intracellular bacteria survival assay

Standard internalization assays were performed. The MOI and time of infection were optimized for each cell type. BMDM (or J774 cells) were culture in 35-mm dishes and exposed to PAK (MOI: 20 for J774). Phagocytosis was allowed for 50 min. Cells were washed with PBS and fresh RPMI supplemented with 400 µg/ml of amikacin and 100 µg/ml of carbenicillin was added for 15 min (or 60 min for J774). Cells were washed again, and media supplemented with 40 µg/ml of amikacin was added and retained throughout the infection. At different intervals, cells were washed and lysed with 0.1% Triton X-100. Serial dilutions of each sample were plated on LB agar in triplicate and incubated overnight at 37 °C. Intracellular survival was determined by CFU counting.

### Endocytic assays

Macrophages were exposed to PAK or AC, or both concurrently, according to the assay. When the Phagocytic Index was determined, the percentage of bacteria internalized per cell was calculated, whether the offered target was PAK alone or in concurrence with AC. Similarly, the Efferocytic Index was calculated as the percentage of apoptotic material internalized per cell, alone or in the concomitant presence of bacteria. Data were normalized to the corresponding controls. The ingested material in each cell was determined by microscopy.

BMDM grown on glass coverslips overnight were exposed to PAK and/or AC for 30 min followed by sample fixation for microscopy analysis. When pre-stimulation treatments were conducted, BMDM were incubated with the different stimuli (PAK, AC, both PAK and AC concurrently, and no stimulus as control) for 50 min. Cells were washed and antibiotic treatment was performed for another 50 min. Then media supplemented with 40 µg/ml of amikacin was added and retained for the 18 h of pre-stimulation. After this, three different possible targets were offered for 50 min: PAK (Fig. 3b), AC (Figs. 3a, 5, 6) or both conjointly (Fig. 3c,d). Endocytic Indexes were determined.

### mRNA isolation, reverse transcription (RT) and qPCR

For macrophage RNA extraction, one million BMDM were seeded into 35-mm tissue culture dishes and exposed to PAK. After 50 min of infection (or LPS treatment, Supplementary Fig. S4c), we proceed with the antibiotic treatment as described above. After a total of 6 h since the initiation of the experiment, cells were washed and total RNA was extracted using Direct-zol RNA Miniprep (Zymo Research, CA, USA), according to the manufacturer’s instructions. Total RNA (500 ng) was retrotranscribed using oligo(dT) and EasyScript RT kit (TransGen Biotech, Beijing, China). qPCR reactions were achieved with TransStart Green qPCR SuperMix (TransGen Biotech) and carried out in a StepOne Plus Real-Time PCR System (Applied Biosystems, Foster City, CA, USA). Reactions were run in triplicates in three independent experiments. For data normalization, we measured mRNA levels for the reference genes *Ywhaz* (tyrosine 3-monooxygenase/tryptophan5-monooxygenase activation protein, zeta polypeptide) and *Ppia* (cyclophilin-a). Normalization with both *Ywhaz* and *Ppia* resulted in almost identical data. Data presented in Fig. 4 correspond to normalization with *Ywhaz*. Relative quantification was performed using a LinReg algorithm^44^.

### Sequences of primers used for qPCR

Primer sequences for reference genes used were: *Ppia*: F 5′-AAGCATACAGGTCCTGGCATCT-3′ and R 5′-CATTCAGTCTTGGCAGTGGCAG-3′, and *Ywhaz* (tyrosine 3-monooxygenase/tryptophan5-monooxygenase activation protein, zeta polypeptide): F 5′-GATGAAGCCATTGCTGAACTTG-3′ and R 5′-GTCTCCTTGGGTATCCGATGTC-3′. The oligonucleotides sequences for other genes measured in this work were: *Il-6*: F 5′-AGAAGGAGTGGCTAAGGACCAA-3′and R 5′-ACGCACTAGGTTTGCCGAGTA-3′, *Il-1β*: F 5′-TCGCTCAGGGTCACAAGAAA-3′ and R5′-AAGGAGGAAAACACAGGCTCTCT-3′, *Tnfα*: F 5′-AGGACCCAGTGTGGGAAGCT-3′ and R 5′-AAAGAGGAGGCAACAAGGTAGAGA, and *Il-10*: F 5′-GCTCCAAGACCAAGGTGTCTACA-3′ and R 5′-GGTGTTTTAGCTTTTCATTTTGATCA-3′.

### IL-6 neutralization assay

0.5 µg/ml of anti-IL-6 (IL-6 Ab) or IgG1 isotype control (IsoAb) antibodies were maintained through the entire experiment. The 50-min pre-stimulation step with bacteria followed by 18 h incubation was performed as described above. Afterwards, labeled AC were offered for determination of the Efferocytic Index. Antibodies were added to the assay in its beginning (following addition of bacterial pre-stimulus), and were renewed as required when washes were performed.

### Microscopy studies

Macrophages were grown on glass coverslips in 24-well plates, at a density of 2 × 10^5^ cells. After the indicated treatments, samples were fixed with 4% paraformaldehyde in PBS for 15 min at room temperature and in the dark, blocked with 2% BSA in PBS and permeabilized with Triton X-100 0.3% (or saponin 0.025% for anti-LAMP1 labelling). When required, they were incubated overnight at 4 °C with the primary antibody, and for 1 h at 37 °C with the secondary antibody and phalloidin. Samples were examined with a confocal laser-scanning microscope Olympus FV1000 using a PlanApo N (60X 1.42 NA) oil objective. Images were taken in the XY plane along the Z axis using a z-step increment of 0.22 μm (Z-stack). Cells were chosen by randomly selecting 5 to 7 fields from each coverslip. All cells in a given field were included in the image analysis. At least 150 cells per condition per experiment were counted.

### Image analysis

Images were analyzed using the Image J program (NIH). To quantify the volume of intracellular apoptotic material or bacteria we used the 3D-Object Counter plugin for ImageJ. This plugin identifies and enumerates fluorescently labeled objects in the z-stack with a user-defined threshold for voxel intensity value. Then, the plugin generates a binary mask showing the particles (adjacent foreground voxels are considered by the program as part of the same particle) and provides a list of the particles with their respective volumes (i.e. number of voxels). Then, the intracellular/extracellular localization of the particles is determined by visual examination (Supplementary Fig. S2). Apoptotic cells are often efferocytosed piecemeal, leaving a window of time during which non-internalized and internalized material are very close to one another. In these cases, the image analysis program may identify these two portions (intra and extracellular) as a single particle. When this is observed during visual determination, we proceed as follows: the zone containing the particle is manually cropped and a more restrictive threshold, that permits the particles to be separated, is applied. Then the corrected volume of the intracellular particle is incorporated in the list.

The number of bacteria per particle was obtained from the ratio between the particle volume and the average volume of one bacterium^14,15^.

When indicated, the confocal image stacks were projected along the z-axis, creating an output image in which each pixel contained the sum over z of the pixel intensity values in that x–y position.

### Statistical analysis

Statistical analyses of the data were performed using GraphPad Prism 6.0 software (CA, USA). Comparisons among three or more groups were assessed with one-way ANOVA, followed by Dunnett’s or Tukey’s multiple comparisons test. Comparisons between two groups were assessed using *t* test analysis. Differences were considered significant if the p values were < 0.05. For the microscopy studies, error bars correspond to standard errors from at least three independent experiments where a minimum of 150 cells were counted per condition.

## Supplementary Information

Supplementary Information

## Abbreviations

AC: Apoptotic cells
BMDM: Bone marrow-derived macrophages
CFSE: Carboxyfluorescein succinimidyl ester
CFU: Colony-forming units
LAMP1: Lysosomal-associated membrane protein 1
LPS: Lipopolysaccharide
M-CSF: Macrophage colony-stimulating factor
MOI: Multiplicity of infection
PAK: *Pseudomonas aeruginosa* Strain K
PAMP: Pathogen-associated molecular patterns
RT: Reverse transcription
TAM receptors: Tyro3, Axl, and Mer receptor tyrosine kinases

## References

[1] Martinez FO, Helming L, Gordon S (2009). Alternative activation of macrophages: An immunologic functional perspective. Annu. Rev. Immunol..

[2] Duffield JS, Lupher M, Thannickal VJ, Wynn TA (2013). Host responses in tissue repair and fibrosis. Annu. Rev. Pathol..

[3] Murray PJ (2017). Macrophage polarization. Annu. Rev. Physiol..

[4] Italiani P, Boraschi D (2014). From monocytes to M1/M2 macrophages: Phenotypical vs. functional differentiation. Front. Immunol..

[5] Mills CD (2012). M1 and M2 macrophages: Oracles of health and disease. Crit. Rev. Immunol..

[6] Atri C, Guerfali FZ, Laouini D (2018). Role of human macrophage polarization in inflammation during infectious diseases. Int. J. Mol. Sci..

[7] Schett G, Neurath MF (2018). Resolution of chronic inflammatory disease: Universal and tissue-specific concepts. Nat. Commun..

[8] Headland SE, Norling LV (2015). The resolution of inflammation: Principles and challenges. Semin. Immunol..

[9] Sugimoto MA, Sousa LP, Pinho V, Perretti M, Teixeira MM (2016). Resolution of inflammation: What controls its onset?. Front. Immunol..

[10] Serhan CN, Savill J (2005). Resolution of inflammation: The beginning programs the end. Nat. Immunol..

[11] Blander JM (2017). The many ways tissue phagocytes respond to dying cells. Immunol. Rev..

[12] Rieber N, Hector A, Carevic M, Hartl D (2014). Current concepts of immune dysregulation in cystic fibrosis. Int. J. Biochem. Cell Biol..

[13] Schwarzer C, Fischer H, Machen TE (2016). Chemotaxis and binding of *Pseudomonas aeruginosa* to Scratch-wounded human cystic fibrosis airway epithelial cells. PLoS One.

[14] Capasso D (2016). Elimination of *Pseudomonas aeruginosa* through efferocytosis upon binding to apoptotic cells. PLoS Pathog..

[15] Lepanto P, Lecumberry F, Rossello J, Kierbel A (2014). A confocal microscopy image analysis method to measure adhesion and internalization of *Pseudomonas aeruginosa* multicellular structures into epithelial cells. Mol. Cell. Probes.

[16] Jawa RS, Anillo S, Huntoon K, Baumann H, Kulaylat M (2011). Analytic review: Interleukin-6 in surgery, trauma, and critical care: Part I: Basic science. J. Intensive Care Med..

[17] Moore KW, de Waal Malefyt R, Coffman RL, O'Garra A (2001). Interleukin-10 and the interleukin-10 receptor. Annu. Rev. Immunol..

[18] Chung EY (2007). Interleukin-10 expression in macrophages during phagocytosis of apoptotic cells is mediated by homeodomain proteins Pbx1 and Prep-1. Immunity.

[19] Cocco RE, Ucker DS (2001). Distinct modes of macrophage recognition for apoptotic and necrotic cells are not specified exclusively by phosphatidylserine exposure. Mol. Biol. Cell.

[20] Campana L (2018). The STAT3-IL-10-IL-6 pathway is a novel regulator of macrophage efferocytosis and phenotypic conversion in sterile liver injury. J. Immunol..

[21] Frisdal E (2011). Interleukin-6 protects human macrophages from cellular cholesterol accumulation and attenuates the proinflammatory response. J. Biol. Chem..

[22] Fernando MR, Reyes JL, Iannuzzi J, Leung G, McKay DM (2014). The pro-inflammatory cytokine, interleukin-6, enhances the polarization of alternatively activated macrophages. PLoS One.

[23] Ferrari E (2017). Cysteamine re-establishes the clearance of *Pseudomonas aeruginosa* by macrophages bearing the cystic fibrosis-relevant F508del-CFTR mutation. Cell Death. Dis..

[24] Mittal R (2016). Otopathogenic *Pseudomonas aeruginosa* enters and survives inside macrophages. Front. Microbiol..

[25] Dacheux D, Goure J, Chabert J, Usson Y, Attree I (2001). Pore-forming activity of type III system-secreted proteins leads to oncosis of *Pseudomonas aeruginosa*-infected macrophages. Mol. Microbiol..

[26] Garai P, Berry L, Moussouni M, Bleves S, Blanc-Potard AB (2019). Killing from the inside: Intracellular role of T3SS in the fate of *Pseudomonas aeruginosa* within macrophages revealed by mgtC and oprF mutants. PLoS Pathog..

[27] Huynh ML, Fadok VA, Henson PM (2002). Phosphatidylserine-dependent ingestion of apoptotic cells promotes TGF-beta1 secretion and the resolution of inflammation. J. Clin. Invest..

[28] Ariel A, Serhan CN (2012). New lives given by cell death: Macrophage differentiation following their encounter with apoptotic leukocytes during the resolution of inflammation. Front. Immunol..

[29] Bratton DL, Henson PM (2011). Neutrophil clearance: When the party is over, clean-up begins. Trends Immunol..

[30] Henson PM (2017). Cell removal: Efferocytosis. Annu. Rev. Cell Dev. Biol..

[31] Fernandez-Boyanapalli RF (2009). Impaired apoptotic cell clearance in CGD due to altered macrophage programming is reversed by phosphatidylserine-dependent production of IL-4. Blood.

[32] Poon IK, Lucas CD, Rossi AG, Ravichandran KS (2014). Apoptotic cell clearance: Basic biology and therapeutic potential. Nat. Rev. Immunol..

[33] Green DR, Oguin TH, Martinez J (2016). The clearance of dying cells: Table for two. Cell Death Differ..

[34] Saas P, Kaminski S, Perruche S (2013). Prospects of apoptotic cell-based therapies for transplantation and inflammatory diseases. Immunotherapy.

[35] Doran AC, Yurdagul A, Tabas I (2020). Efferocytosis in health and disease. Nat. Rev. Immunol..

[36] Xing Z (1998). IL-6 is an antiinflammatory cytokine required for controlling local or systemic acute inflammatory responses. J. Clin. Invest..

[37] Mukherjee S (2017). Boosting efferocytosis in alveolar space using BCG vaccine to protect host against influenza pneumonia. PLoS One.

[38] Bleriot C (2015). Liver-resident macrophage necroptosis orchestrates type 1 microbicidal inflammation and type-2-mediated tissue repair during bacterial infection. Immunity.

[39] Fujimori T (2015). The Axl receptor tyrosine kinase is a discriminator of macrophage function in the inflamed lung. Mucosal Immunol..

[40] Grabiec AM, Goenka A, Fife ME, Fujimori T, Hussell T (2018). Axl and MerTK receptor tyrosine kinases maintain human macrophage efferocytic capacity in the presence of viral triggers. Eur. J. Immunol..

[41] Zagorska A, Traves PG, Lew ED, Dransfield I, Lemke G (2014). Diversification of TAM receptor tyrosine kinase function. Nat. Immunol..

[42] Marim FM, Silveira TN, Lima DS, Zamboni DS (2010). A method for generation of bone marrow-derived macrophages from cryopreserved mouse bone marrow cells. PLoS One.

[43] Lagendijk EL, Validov S, Lamers GE, de Weert S, Bloemberg GV (2010). Genetic tools for tagging Gram-negative bacteria with mCherry for visualization in vitro and in natural habitats, biofilm and pathogenicity studies. FEMS Microbiol. Lett..

[44] Ruijter JM (2013). Evaluation of qPCR curve analysis methods for reliable biomarker discovery: Bias, resolution, precision, and implications. Methods.

