# Supplementary material for: The inflammatory response induced by Pseudomonas aeruginosa in macrophages enhances apoptotic cell removal
Source: Sci Rep. 2021 Jan 27;11:2393. doi: 10.1038/s41598-021-81557-1 (PMC7841155; doi:10.1038/s41598-021-81557-1)
Supplement: Supplementary file 1 — Supplementary Information [file 41598_2021_81557_MOESM1_ESM.pdf]

Supplementary Info:

The inflammatory response induced by *Pseudomonas aeruginosa* in macrophages enhances apoptotic cell removal

Adriana Valeria Jäger *†, Paula Arias †, Maria Virginia Tribulatti, Marcela Adriana Brocco, Maria Victoria Pepe, Arlinet Kierbel *

Instituto de Investigaciones Biotecnológicas "Dr. R. Ugalde", IIBIO, Universidad Nacional de San Martín (UNSAM), CONICET

† These authors contributed equally to this work.

* These authors are co-senior authors.

Correspondence: A.V. Jäger and A. Kierbel, Instituto de Investigaciones Biotecnológicas (IIBIO), Universidad Nacional de San Martín, Buenos Aires B1650HMP, Argentina.

Correspondence: avjager@gmail.com; akierbel@iibintech.com.ar

a

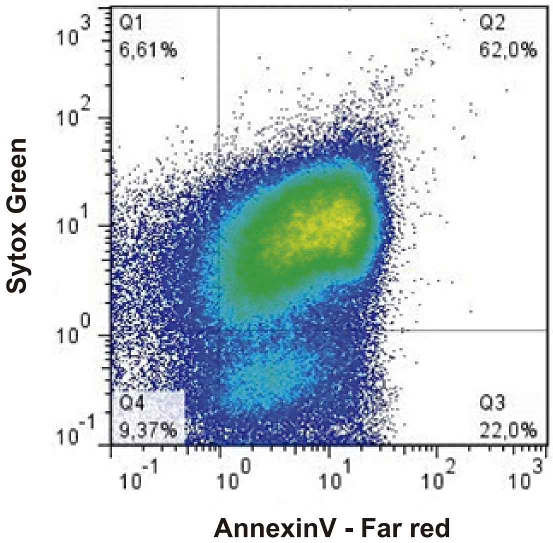

b

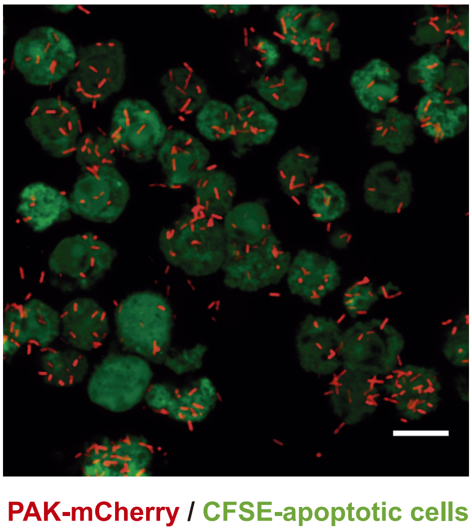

Supplementary Figure S1. ***P. aeruginosa adheres to J774 apoptotic cells.*** **a.** Flow cytometry analysis of Annexin V-Sytox Green double staining of UV-treated J774 cells. On the flow cytometric scatter graph, the right upper quadrant represents our population of interest: the late apoptotic cells (Q2: 62%). The right lower quadrant represents the early apoptotic cells (Q3: 22%). **b.** Projected confocal Z-stack of apoptotic J774 co-incubated with bacteria for one hour. Almost all bacteria were found adhered to dead cells with few free bacteria remaining. Green: CFSE-labeled apoptotic cells. Red: PAK-mCherry.

a

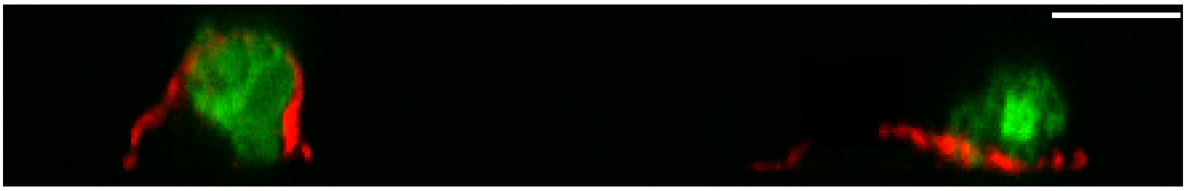

b

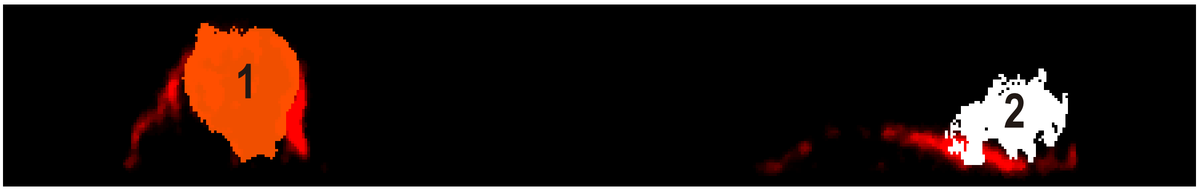

c

Particle #	Volume (μm^3)	Volume (voxels)	Position
1	44	4705	Intracellular
2	26	2846	Extracellular

Supplementary Figure S2: ***Image analysis.*** Representative image showing how the Object counter tool from ImageJ is used to evaluate the volume of BMDM-associated apoptotic cell material. **a.** CFSE-labeled apoptotic cells (green) associated with phalloidin-labeled cells (red). Scale bar: 10 µm. **b.** “Object or particle map” rendered by the Object counter tool. **c.** A chart listing the volume (in voxels and μm^3) of the particles. The localization (i.e. extracellular or intracellular) of apoptotic material was defined visually.

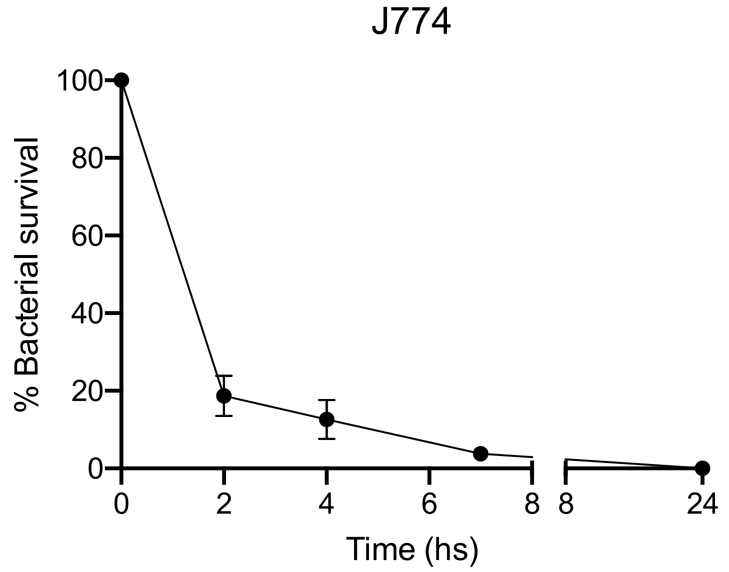

Supplementary Figure S3: *P. aeruginosa is eliminated by J774 macrophage-like cell line.* Intracellular bacterial survival over time was measured by standard antibiotic protection assays. J774 were infected with a MOI of 20 for 60 min, followed by 60 min of antibiotic treatment. Subsequently intracellular survival was determined by the CFU method at different time points. The mean of CFU ± SEM was calculated. Data were normalized to time 0 (100%). Three independent experiments were performed.

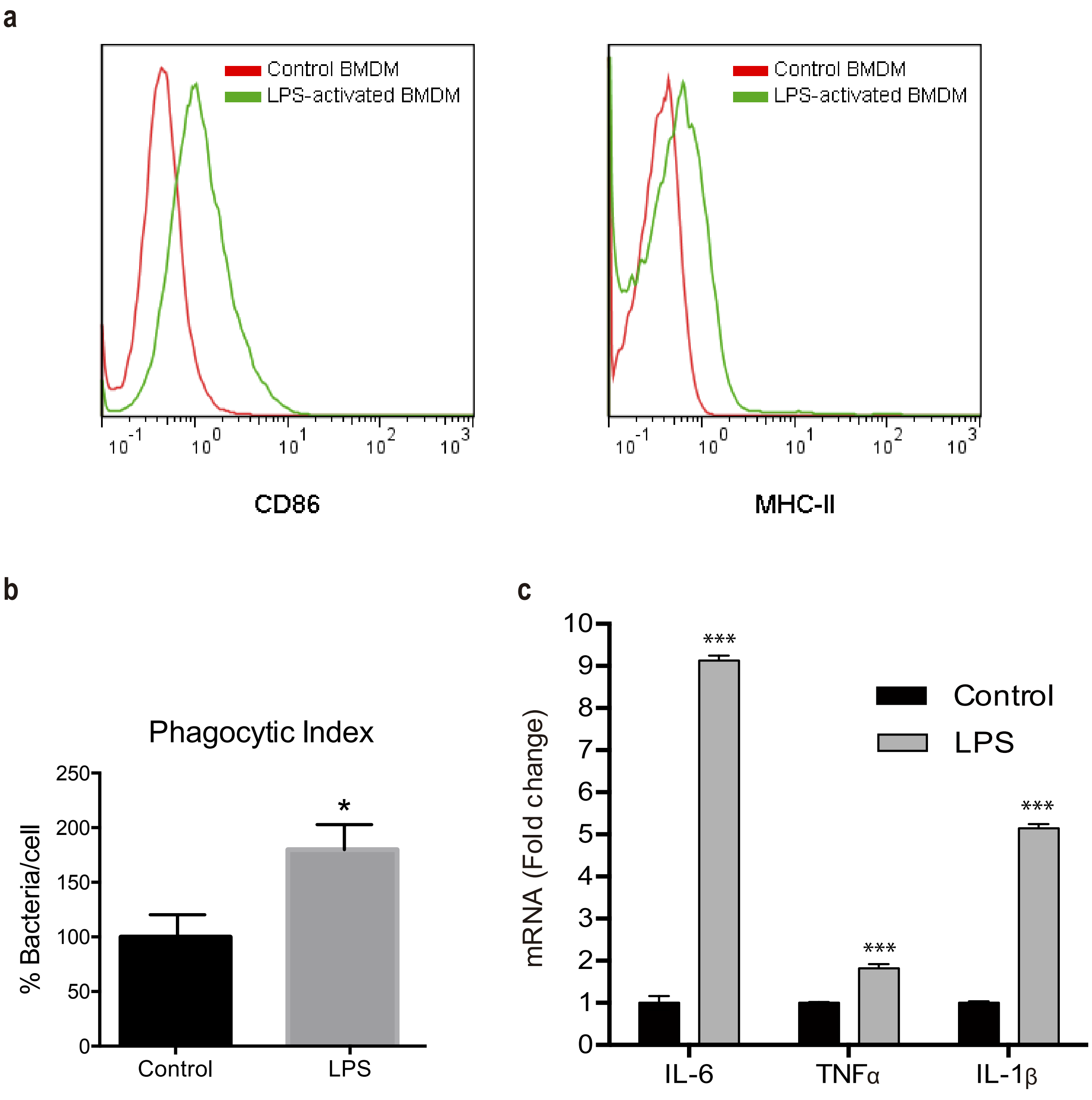

Supplementary Figure S4: ***BMDM responsiveness to LPS inflammatory stimulus.*** **a.** CD86 and MHC-II expression on BMDM surface after LPS-stimulation. BMDM were incubated for one hour with LPS (1 µg/ml) or left untreated (Control) and surface molecule expression was determined by flow cytometry. Representative histogram plots are shown. **b.** BMDM Phagocytic Index is increased by LPS pre-stimulation. BMDM were incubated for 50 min with LPS (500 ng/ml) or left untreated (Control). The phagocytic capacities were checked 18 hours after pre-stimuli. Data were normalized to the Control (non-stimulus: 100%) and expressed as mean of bacteria/cell ± SEM. * p=0.0357 *vs.* Control by unpaired *t*-test. **c.** Cytokine gene expression was determined in BMDM after stimulus with LPS and expressed as a fold change with respect to Control (non-stimulus). BMDM were incubated for 50 min with LPS (100 ng/ml) or left untreated (Control). Six hours after the initiation of the experiment, RNA was extracted. Three pro-inflammatory cytokines were tested by RT-qPCR: *Il-6, TNFα and Il-1β*. Data are presented as mean Fold Change ± SD and were normalized to *Ywhaz* mRNA levels. Reactions were run in triplicates. Significant differences were found for the three cytokines between LPS-treated and Control groups (*** p≤0.0003 by unpaired *t*-test).
